# A simplified prediction model for end-stage kidney disease in patients with diabetes

**DOI:** 10.1038/s41598-022-16451-5

**Published:** 2022-07-21

**Authors:** Toyoshi Inoguchi, Tasuku Okui, Chinatsu Nojiri, Erina Eto, Nao Hasuzawa, Yukihiro Inoguchi, Kentaro Ochi, Yuichi Takashi, Fujiyo Hiyama, Daisuke Nishida, Fumio Umeda, Teruaki Yamauchi, Daiji Kawanami, Kunihisa Kobayashi, Masatoshi Nomura, Naoki Nakashima

**Affiliations:** 1 Fukuoka City Health Promotion Support Center, Fukuoka City Medical Association, Maizuru 2-5-1, Chuou-ku, Fukuoka, 810-0073 Japan; 2 Medical Information Center, Kyushu University Hospital, Fukuoka, 812-8582 Japan; 3 Department of Diabetes and Endocrinology, Saga-Ken Medical Centre Koseikan, Saga, 840-8571 Japan; 4 Division of Endocrinology and Metabolism, Department of Internal Medicine, Kurume University School of Medicine, Kurume, 830-0011 Japan; 5 Yukuhashi Central Hospital, Yukuhashi, 824-0031 Japan; 6 Department of Endocrinology and Diabetes Mellitus, Fukuoka University Chikushi Hospital, Chikushino, 818-8502 Japan; 7 Department of Endocrinology and Diabetes Mellitus, School of Medicine, Fukuoka University, Fukuoka, 814-0180 Japan; 8 Carna Health Support, Co., Ltd., Fukuoka, 810-0054 Japan

**Keywords:** Biomarkers, Endocrinology, Nephrology, Risk factors

## Abstract

This study aimed to develop a simplified model for predicting end-stage kidney disease (ESKD) in patients with diabetes. The cohort included 2549 individuals who were followed up at Kyushu University Hospital (Japan) between January 1, 2008 and December 31, 2018. The outcome was a composite of ESKD, defined as an eGFR < 15 mL min^−1^ [1.73 m]^−2^, dialysis, or renal transplantation. The mean follow-up was 5.6 $\pm$ 3.7 years, and ESKD occurred in 176 (6.2%) individuals. Both a machine learning random forest model and a Cox proportional hazard model selected eGFR, proteinuria, hemoglobin A1c, serum albumin levels, and serum bilirubin levels in a descending order as the most important predictors among 20 baseline variables. A model using eGFR, proteinuria and hemoglobin A1c showed a relatively good performance in discrimination (C-statistic: 0.842) and calibration (Nam and D’Agostino $\chi$^2^ statistic: 22.4). Adding serum albumin and bilirubin levels to the model further improved it, and a model using 5 variables showed the best performance in the predictive ability (C-statistic: 0.895, $\chi$^2^ statistic: 7.7). The accuracy of this model was validated in an external cohort (n = 5153). This novel simplified prediction model may be clinically useful for predicting ESKD in patients with diabetes.

## Introduction

Diabetic kidney disease (DKD) continues to be the leading cause of end-stage kidney disease (ESKD) and accounts for $\ge$ 40% of patients receiving dialysis and renal transplantation in many countries^1,2^. Early and accurate prediction of progression to ESKD is of practical benefit in patients with diabetes.

In current clinical practice, the estimated glomerular filtration rate (eGFR) and the presence of proteinuria (albuminuria) are used as the main predictors for progression of chronic kidney disease (CKD) including DKD^3,4^. However, these two variables may not be sufficient for clinical decision-making, especially in DKD. In fact, other clinical variables, such as glycemic control, affect the progression of DKD^5,6^. Various prediction models for the progression of CKD to ESKD have been reported^7–10^, and some ESKD prediction equations are widely used through electronic applications^10^. However, CKD is heterogenous in the variability of progression rates and its pathogeneses. Therefore, developing a prediction model specific for DKD is important. None of the CKD models include variables related to glycemic control such as hemoglobin A1c (HbA1c) levels. Therefore, several prediction models have also been reported for predicting ESKD in people with diabetes^11–15^. These models showed a moderate to good performance for predicting ESKD, but they used many predictive variables in addition to the eGFR (or creatinine) and albuminuria. For clinical use, the ideal prediction models should not only be accurate, but also easy to implement. In this study, we aimed to develop a simplified, but accurate model for predicting ESKD in patients with diabetes using a few commonly available variables that are easy to measure in the primary care setting.

In recent years, oxidative stress has been considered to be an important pathogenic factor in the development of DKD^16–21^. Since bilirubin and albumin are potent endogenous antioxidants in serum^22–24^, these variables may play an important role in the progression of DKD^10,11,14,15,25–28^. Therefore, in this study, we first evaluated the relative importance of various possibly predictive variables including serum bilirubin and albumin levels for ESKD using a machine learning random forest model. The benefit of the machine learning approach is that it can use a data-driven approach to analyze a large number of variables. We then developed a final simplified prediction model using the minimum number of selected important variables and Cox proportional hazard model, which may be useful for predicting ESKD in primary care. We also validated the performance of this model in an independent external cohort.

## Results

### Characteristics of study subjects in the development cohort

A total of 2,549 patients (1,432 men and 1,117 women) were eligible for inclusion in the analysis. Table 1 shows the baseline characteristics of the enrolled patients. The median age was 57 years (interquartile range [IQR]: 47–63), and the mean follow-up was 5.6 $\pm$ 3.7 years. There were 176 ESKD events (6.2%) during the follow-up. The median time to ESKD was 2.5 years (IQR: 0.9–4.8).

**Table 1:** Baseline characteristics of study subjects (n = 2549).

**Variables**	
Age, years, median (IQR)	57.0 (47.0–63.0)
Gender, male, n (%)	1432 (56.2)
**Smoking, n (%)**	
Smoker	1133 (44.4)
Missing	527 (20.7)
Body mass index, kg/m^2^, median (IQR)	24.2 (21.7–27.4)
Hypertension, n (%)	1462 (57.4)
**Dyslipidemia, n (%)**	
Positive	1836 (72.0)
Missing	15 (0.6)
HbA1c, %, median (IQR)	7.0 (6.5–8.1)
mmol/mol, median (IQR)	53.0 (47.5–65.0)
Serum albumin, mg/dL, mean (SD)	4.0 (0.5)
Serum bilirubin, mg/dL, median (IQR)	0.7 (0.5–0.9)
Serum uric acid, mg/dL, mean (SD)	5.3 (1.5)
White blood cells, × 10^3^/mL, mean (SD)	7.21 (2.26)
Red blood cells, × 10^6^/mL, mean (SD)	4.44 (0.57)
Thrombocytes, × 10^4^/mL, mean (SD)	23.4 (6.9)
eGFR, mL min^−1^ [1.73 m]^−2^, mean (SD)	81.8 (26.6)
**Proteinuria, n (%)**	
Positive	475 (29.7)
Missing, n (%)	948 (37.2)
Statin use, n (%)	865 (33.9)
Fibrate-related drug use, n (%)	54 (2.1)
ARB use, n (%)	785 (30.8)
ACE inhibitor use, n (%)	263 (10.3)
Erythropoiesis stimulating agent use, n (%)	42 (1.6)
GLP-1R agonist use, n (%)	62 (2.4)
SGLT2 inhibitor use, n (%)	51 (2.0)
Metformin use, n (%)	517 (20.3)
**Follow-up time, years**	
Median (IQR)	4.7 (2.3–8.8)
Mean (SD)	5.6 (3.7)
ESKD, n (%)	176 (6.2)
**Time to ESKD, years**	
Median (IQR)	2.5 (0.9–4.8)
Mean (SD)	3.2 (2.9)

Data are presented as the mean $\pm$standard deviation (SD) or the median (interquartile range: IQR).

*HbA1c* hemoglobin A1c, *eGFR* estimated glomerular filtration rate, ARB angiotensin II receptor blocker, *ACE* angiotensin converting enzyme, *GLP-1R* glucagon-like peptide 1 receptor, *SGLT2* sodium-glucose cotransporter 2, *ESKD* end-stage kidney disease.

### Performance of prediction models in the development cohort

The random forest model using 20 variables showed an excellent predictive ability for ESKD (Harrell’s concordance statistic [C-statistic]; 0.935), and selected eGFR, proteinuria, HbA1c, serum albumin, and serum bilirubin as the most important predictors in descending order (Table 2). The Cox proportional hazard model also showed a similar performance in predictive ability (C-statistic: 0.905) and the upper 5 variables were the same as those in the random forest model (Table 2). Therefore, we developed a sequential series of models using these 5 selected variables and Cox proportional hazard models, and then compared their performances. The hazard ratios for the variables and C-statistics for the models are shown in Table 3. The C-statistic was 0.736 for Model 1 (eGFR alone), 0.806 for Model 2 (eGFR and proteinuria), 0.841 for Model 3 (eGFR, proteinuria, and HbA1c), 0.852 for Model 4.1 (eGFR, proteinuria, HbA1c, and serum albumin), 0.881 for Model 4.2 (eGFR, proteinuria, HbA1c, and serum bilirubin), and 0.895 for Model 5 (eGFR, proteinuria, HbA1c, serum albumin, and serum bilirubin). Comparing with the basic model (Model 2, eGFR and proteinuria), the C statistic was significantly increased in Model 3 and Model 5 (P = 0.030, P = 0.012, respectively), suggesting that Model 3 and 5 have a significantly better performance in the discrimination of ESKD than Model 2. In addition, there was no significant difference in the C statistic between these Models and Model 6 (all 20 variables), suggesting that Model 3 and 5 are highly efficient in the discrimination. Next, the prediction risk scores for ESKD using Models 3 and 5 from the Cox proportional hazard model were calculated as follows:

$${\text{Risk score }}\left( {\text{Model 3}} \right)\, = \,\left( { - \,0.0{59}\, \times \,{\text{eGFR in mL min}}^{{ - {1}}} \left[ {{1}.{73} {\text{m}}} \right]^{{ - {2}}} } \right)\, + \,\left( {0.{415}\, \times \,{\text{HbA1c in }}\% } \right)\, + \,({1}.{822}\, \times \,{\text{1 if positive for proteinuria}}).$$

$${\text{Risk score }}\left( {\text{Model 5}} \right)\, = \,\left( { - \,0.0{52}\, \times \,{\text{eGFR in mL min}}^{{ - {1}}} \left[ {{1}.{73} {\text{m}}} \right]^{{ - {2}}} } \right)\, + \,\left( {0.{368}\, \times \,{\text{HbA1c in }}\% } \right)\, + \,\left( { - \,0.{972}\, \times \,{\text{albumin levels in mg}}/{\text{dL}}} \right)\, + \,\left( { - \,{1}.{41}0\, \times \,{\text{bilirubin levels in mg}}/{\text{dL}}} \right)\, + \,({1}.{27}0\, \times \,{\text{1 if positive for proteinuria}}).$$

**Table 2:** Relative importance of variables for predicting end-stage kidney disease (ESKD) using the random forest model and Cox proportional hazard model.

Random forest model C-statistic 0.935			
Upper 12 variables	Relative importance		
eGFR	0.083		
Proteinuria, positive	0.027		
HbA1c	0.022		
Serum albumin	0.020		
Serum bilirubin	0.006		
Serum uric acid	0.005		
Red blood cell count	0.004		
ARB use	0.003		
Age	0.002		
Hypertension, positive	0.001		
Body mass index	0.001		
Thrombocyte count	0.000		

Cox proportional hazard model C-statistic 0.905			
Upper 12 variables	Hazard ratio	95% CI	*P* value
eGFR	0.248	0.194–0.315	< 0.001
HbA1c	1.806	1.572–2.074	< 0.001
Proteinuria, positive	1.612	1.304–1.992	< 0.001
Serum albumin	0.603	0.505–0.720	< 0.001
Serum bilirubin	0.648	0.492–0.854	0.002
Age	0.656	0.547–0.787	< 0.001
ARB use	1.276	1.040–1.566	0.020
GLP-1R agonist use	0.819	0.678–0.991	0.040
Serum uric acid	1.164	0.967–1.401	0.109
Hypertension, positive	0.848	0.640–1.123	0.250
Gender, men	1.135	0.946–1.361	0.174
Smoking, positive	1.125	0.944–1.342	0.189

*eGFR* estimated glomerular filtration rate, *HbA1c* hemoglobin A1c, *ARB* angiotensin II receptor blocker, *GLP-1R* glucagon-like peptide 1 receptor.

**Table 3:** Hazard ratios and C-statistics of various models for predicting end-stage kidney disease (ESKD) as evaluated by the Cox proportional hazard model.

Model	Explanation variables	Hazard ratio	95% CI Lower–upper	*P* value	C-statistic (SD)
1	eGFR, per 5 mL min^−1^ [1.73 m]^−2^	0.690	0.663–0.717	< 0.001	0.736 (0.091)
2	eGFR, per 5 mL min^−1^ [1.73 m]^−2^ Proteinuria, positive	0.754 6.679	0.724–0.785 4.443–10.038	< 0.001 < 0.001	0.806 (0.090)
3	eGFR, per 5 mL min^−1^ [1.73 m]^−2^ Proteinuria, positive HbA1c, per 1%	0.745 6.755 1.493	0.716–0.776 4.421–10.321 1.379–1.615	< 0.001 < 0.001 < 0.001	0.841 (0.068)
4.1	eGFR, per 5 mL min^−1^ [1.73 m]^−2^ Proteinuria, positive HbA1c, per 1% Serum albumin, per 3 mg/dL	0.763 4.106 1.460 0.581	0.733–0.795 2.610–6.458 1.349–1.579 0.507–0.665	< 0.001 < 0.001 < 0.001 < 0.001	0.852 (0.070)
4.2	eGFR, per 5 mL min^−1^ [1.73 m]^−2^ Proteinuria, positive HbA1c, per 1% Serum bilirubin, per 0.1 mg/dL	0.766 6.199 1.443 0.797	0.736–0.798 4.033–9.528 1.336–1.560 0.732–0.867	< 0.001 < 0.001 < 0.001 < 0.001	0.881 (0.061)
5	eGFR, per 5 mL min^−1^ [1.73 m]^−2^ Proteinuria, positive HbA1c, per 1% Serum albumin, per 3 mg/dL Serum bilirubin, per 0.1 mg/dL	0.773 4.019 1.432 0.626 0.865	0.742–0.805 2.540–6.361 1.325–1.548 0.543–0.722 0.797–0.938	< 0.001 < 0.001 < 0.001 < 0.001 < 0.001	0.895 (0.065)
6	All variables				0.905 (0.050)

*eGFR* estimated glomerular filtration rate, *HbA1c* hemoglobin A1c, *SD* standard deviation.

Using these risk scores, the 5-year risk of each individual in Models 3 and 5 was estimated as follows:

$$ \begin{gathered} {\text{5 - year risk }}\left( {\text{Model 3}} \right):p\left( {{1},{825}} \right) \, = {1} - {\text{ exp }}\left( { - H_{0} \left( {{1},{825}} \right)} \right)^{{{\text{exp }}({\text{risk score in Model 3}})}} \, \hfill \\ = \,{1}{-}{\text{ exp }}\left( { - \,0.0{43}} \right)^{{{\text{exp }}({\text{risk score in Model 3}})}} \, = \,{1} \hfill \\ \hfill \\ {\text{5 - year risk }}\left( {\text{Model 5}} \right):p\left( {{1},{825}} \right)\, = \,{1}{-}{\text{exp }}\left( { - \,H_{0} \left( {{1},{825}} \right)} \right)^{{{\text{exp }}({\text{risk score in Model 5}})}} \hfill \\ = \,{1}{-}{\text{ exp }}\left( { - \,{5}.{26}0} \right)^{{{\text{exp }}({\text{risk score in Model 5}})}} \, = \,{1}  \hfill \\ \hfill \\ \end{gathered} $$

**Figure 1:**
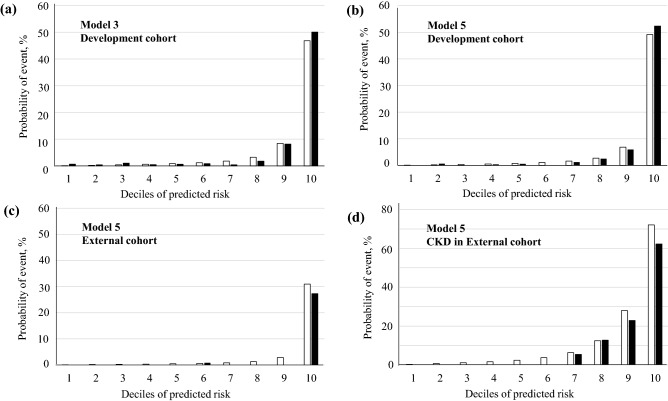
Observed vs predicted probabilities of end-stage kidney disease (ESKD) events at a 5-year risk in the development cohort and the external validation cohort. The predicted (white bar) and observed (black bar) event probabilities represent the mean predicted probability calculated from 5-year risk equations and the mean observed probability from the patients divided into deciles of the predicted probability, respectively. (**a**) Model 3 and (**b**) Model 5 in the development cohort. (**c**) Model 5 in the validation cohort and (**d**) Model 5 in the patients with chronic kidney disease (CKD) (eGFR < 60 mL min^−1^ [1.73 m]^−2^ and/or positive proteinuria) (n = 1350) of the validation cohort. Nam and D’Agostino $\chi$^2^ statistics were 22.4 and 7.7 for Models 3 and 5 in the development cohort, and 36.1 and 23.1 for Models 5 in the external validation cohort and Model 5 in the patients with CKD of the external cohort, respectively.

**Figure 2:**
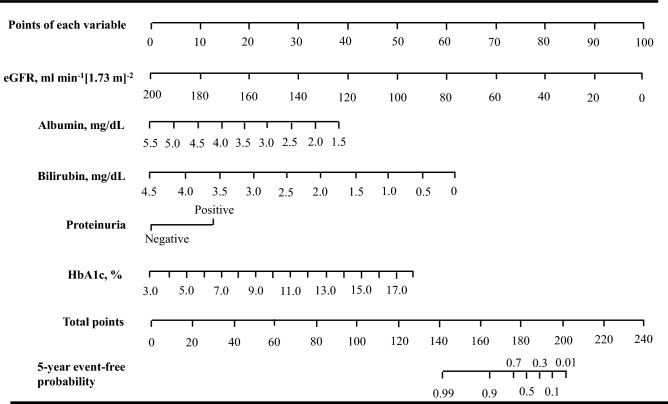
Nomogram for end-stage kidney disease (ESKD)-free event probabilities of individuals with diabetes. To use the nomogram. Locate an individual’s value on each variable axis, and draw a line upward to obtain the point for each variable. Then, locate the sum of these points on the total points axis, and draw a line downward to the event-free axis to obtain the 5-year ESKD-free probability. *eGFR* estimated glomerular filtration rate, *HbA1c* hemoglobin A1c.

Next, calibration was examined by comparing the observed vs predicted probabilities of ESKD at a 5-year risk for Models 3 and 5 (Fig. 1a,b). The predicted probabilities were calculated from the above 5-year risk equations for Models 3 and 5. The Nam and D’Agostino $\chi$^2^ statistics were 22.4 and 7.7 for Models 3 and 5, respectively. These findings suggested that the calibration showed an appropriate agreement between observed vs predicted probabilities for both Models, but Model 5 was more accurate than Model 3. Therefore, we built the nomogram to predict the ESKD probability for each individual based on the Model 5 (Fig. 2), which may provide a practical tool for clinical application.

### Performance of prediction models in the external validation cohort

A total of 5153 patients were eligible for inclusion in the validation analysis. The baseline characteristics were shown in supplementary Table S1. The baseline risk in this cohort appeared to differ from those in the development cohort. The patients in the external cohort had complete data for the presence of proteinuria, but the rate of proteinuria was still lower in the external cohort than that in the development cohort with missing data (19.1% vs 26.6%). The rate of ESKD events in the external cohort was lower than that in the development cohort (3.1% vs 6.2%, *P* < 0.001). Despite the difference in baseline characteristics, the predictive performance of Model 5 was excellent in discrimination (C-statistic: 0.951) in the external cohort. In calibration, its performance was moderate without recalibration ($\chi$^2^ statistic: 36.1 at a 5-year risk) (Fig. 1c) and there was a tendency to slightly overestimate risk. This was likely to be due to the lower baseline risk in the external cohort than that in the development cohort. For the high-risk subgroup of the individuals with CKD in the external cohort (eGFR < 60 mL min^−1^ [1.73 m]^−2^ and/or positive proteinuria) (n = 1,350), its performance was good in discrimination (C-statistic: 0.862) and adequate in calibration without recalibration ($\chi$^2^ statistic: 23.1 at a 5-year risk) (Fig. 1d), suggesting the clinical usefulness of this model for predicting ESKD.

## Discussion

In this study, we first evaluated the relative importance of 20 possibly predictive variables for ESKD using a machine learning random forest model and a Cox proportional hazard model. Both models selected eGFR, proteinuria, hemoglobin HA1c, serum albumin, and serum bilirubin as the most important predictors. Then, we developed a simplified prediction model using these 5 variables and the Cox proportional hazard model. To the best of our knowledge, this is the first report to show that a 5-year risk model using these 5 commonly available variables has a good performance in the predictive ability for ESKD in patients with diabetes.

Previously, several prediction models for ESKD in patients with diabetes have also been reported^11–15^. Jardine et al. reported a prediction model using 7-variables, including eGFR, urinary albumin-creatinine ratio, sex, systolic blood pressure, blood pressure-lowering agent use, presence of retinopathy, and education career from the ADVANCE trial (C-statistic: 0.847)^12^. A similar prediction model using 11 variables was reported in Chinese patients with diabetes (area under the curves [AUC] of the 3-, 5-, and 8-year risk: 0.90, 0.86, and 0.81, respectively)^14^. However, these models lacked external validation and thereby may not be generalized well to other populations. The model reported by Elley et al. showed a good performance in the predictive ability in the development cohort and the external validation cohort (C-statistic: 0.89–0.92), but this model used 10 variables including sex, ethnicity, age, diabetes duration, albuminuria, serum creatinine, systolic blood pressure, HbA1c, smoking status, and previous cardiovascular disease status^13^. A recent study developed a machine learning based prediction model called the feed-forward neural network model^15^. In that model, 18 variables were used in patients with diabetes and nephropathy participating in past clinical trials, including RENAAL, IDNT and ALTITUDE studies (AUC: 0.82, 0.81, and 0.84, respectively). The machine learning approach appears to be superior to the traditional hypothesis-driven statistical methods in terms of its data-driven approach to analyze a large number of possibly predictive variables. Our random forest model using 20 variables also showed an excellent predictive ability for ESKD (C-statistic 0.935). However, the main obstacle is that many predictive variables are not readily obtainable in primary care, thus limiting their usefulness to clinicians’ managing patients with diabetes. In contrast, Keane et al. reported a simple prediction model using four variables (serum creatinine, urine albumin-creatinine ratio, serum albumin, and hemoglobin) in a cohort from the RENNAL study^11^. They selected those four variables from 23 baseline variables using the Cox proportional hazard model with backward selection process, with *P* < 0.01 required for inclusion in a final model. However, our analysis using both the machine learning approach and the Cox proportional hazard model showed that HbA1c levels and bilirubin levels were more important predictors than hemoglobin levels for predicting ESKD. The mean follow-up period was much shorter in the RENNAL study than in our study (3.4 years vs. 5.6 years), and decreasing hemoglobin level is a generally late sign of renal impairment. Their model may be effective for risk prediction at a time shorter than 3 years. Thus, our simplified 5-year prediction model may be more useful than previous prediction models in clinical practice. Our model could guide clinicians in making clinical decision earlier regarding intensification of monitoring and preventive therapies or referral to specialists. Risk information helps patients to become aware of their current risk, promote motivation on improving their lifestyle. In our study, the nomogram based on our model was built to predict the absolute ESKD probability for each individual. The 5-year risk equation we showed can be also used as electronic applications. These tools may provide practical risk predictive tools for future clinical application.

The reason why serum bilirubin levels were so important among various predictive variables for predicting ESKD remained to be elucidated. Bilirubin is a product of heme catabolism by heme oxygenase, which is a major antioxidant enzyme. Bilirubin is thought to have a protective effect on oxidative stress-induced organ damage through its strong antioxidant activity^22^. We previously showed a lower prevalence of nephropathy and other vascular complications, as well as reduced oxidative stress, in patients with diabetes and Gilbert syndrome, which is a hereditary hyperbilirubinemia^25^. Accumulating evidence has also shown that serum bilirubin levels are negatively associated with the progression of DKD^26–28^. Taken together, it is most likely that serum bilirubin may prevent the progression of nephropathy via its anti-oxidative activities. This possibility is supported by an animal study, which showed that bilirubin prevented renal oxidative stress and dysfunction in type-1 diabetic rats and type 2 diabetic mice^29^. In addition, serum bilirubin levels have been reported to be affected by oxidative stress-related factors such as smoking, obesity, hypertension, metabolic syndrome, and cardiovascular diseases in addition to genetic factors^30–34^, all of which are possible risk facors for DKD. Therefore, serum bilirubin levels might represent a total susceptibility determined by such factors to the progression to ESKD. In line with these concepts, the effect of anti-oxidative properties of albumin on the progression to ESKD may be plausible, although the effect of serum albumin levels may be mainly explained by their association with the levels of albuminuria. Albumin is thought to be an important serum antioxidant in addition to serum bilirubin^24,25^. In serum, free thiol groups are one of the most important scavengers of hydroxy radicals and other oxidants and are largely located on albumin^35^. Serum albumin levels have been reported to be inversely associated with the cardiovascular disease risk and aging, supporting its possible causal relationships with the oxidative stress-related status and diseases^36–38^. The mechanisms underlying the close associations of serum bilirubin and albumin levels with the progression to ESKD should be clarified in future studies.

There are several limitations to this study. First, we might not have obtained ideal information regarding clinical data and timely assessment of endpoint, compared with controlled clinical trials. Second, the sample size may not have been sufficient to develop prediction models. Third, we used proteinuria by a conventional urine test rather than measurements of albuminuria because the rate of albuminuria measurements was low in the electronic medical record data, although proteinuria data are much more easily obtainable than those of albuminuria measurement in primary care. Forth, a competitive risk analysis of death was not performed because only 16 death cases occurred and the relationship between each death and kidney dysfunction was unknown in this study using electronic medical records. Despite these limitations, our prediction models showed a good performance in the independent external validation as well as the development cohorts. Lastly, although the excellent performance in discrimination of our prediction model was confirmed in an external cohort, the performance in calibration should be evaluated in more various populations including different ethnicities, and cohorts outside Japan for widespread adoption of this model, because patient characteristics, healthcare system, and treatment strategies vary between health centers, regions and countries, and such heterogeneity can affect risk estimates and their calibration^39,40^.

In conclusion, we developed a simplified and accurate 5-year prediction model using the commonly available clinical variables of eGFR, proteinuria, HbA1c, serum albumin and bilirubin levels, which are easy to measure. Prospective studies should be done to establish its clinical usefulness in reducing ESKD in patients with diabetes.

## Methods

### Study subjects in the development cohort

We obtained data from the electronic medical record system at Kyushu University Hospital (Japan) between January 1, 2008 and December 31, 2018. Eligible patients with diabetes were aged 20–69 years and had laboratory data, including the eGFR, and serum bilirubin and albumin levels at baseline. In this study, diabetes was defined as casual blood glucose levels $\ge$ 200 mg/dL, HbA1c levels $\ge$ 6.5%, or the use of glucose lowering agents. Other inclusion criteria were a follow-up $\ge$ 1 year, and at least 5 measurements of the eGFR during follow-up. Patients were excluded if they had an eGFR $\le$ 15 mL min^−1^ [1.73 m]^−2^, received dialysis or renal transplantation at baseline. Other exclusion criteria were co-occurrence of acute kidney injury or cancer during follow-up, or co-occurrence of liver cirrhosis, other hepatobiliary diseases with abnormal liver enzyme levels (alanine aminotransferase or alkaline phosphatase levels $\ge$ 2 fold of the upper limit of the normal range), or hemolytic anemia to evaluate the true contribution of serum bilirubin levels to ESKD events. Patients with missing data were also excluded in this analysis if the rate of missing data was very low (< 3%). A detailed flowchart of the patients’ selection is shown in the supplementary Fig. S1.

### Study subjects in the validation cohort

For the independent external validation cohort, we obtained data from the medical record data for patients (n = 5153) who were followed up at 5 hospitals or one disease management institute from January 1, 2008 to December 31, 2020 and had laboratory data, including the eGFR, HbA1c levels, proteinuria, serum albumin levels, and bilirubin levels, at baseline. The same exclusion criteria as those in the development cohort were used.

All procedures were performed in accordance with the relevant guidelines and regulations. Informed consent was obtained from all participants and/or their legal guardians. The study was approved by the ethics committees of Kyushu University Hospital and other related institutes.

### Variables

Twenty variables were used to select important variables for the prediction model for ESKD, including age, sex, body mass index (BMI), smoking status, presence of hypertension, presence of dyslipidemia, eGFR, HbA1c, serum albumin, serum bilirubin, serum uric acid, red blood cell count, white blood cell count, platelet count, presence of proteinuria, and medication use (angiotensin II converting enzyme inhibitors, angiotensin II receptor blockers, statins, fibrate-related drugs, and GLP-1 receptor agonists) (Table 1). eGFR was calculated with an equation from the Japanese Society of Nephrology^41^. HbA1c levels were presented as the National Glycohemoglobin Standardization Program value and the International Federation of Clinical Chemistry and Laboratory Medicine mmol/mol units converted using the National Glycohemoglobin Standardization Program converter for HbA1c, available at http://www.ngsp.org/convert1.asp. The presence of hypertension was based on the ICD code. Dyslipidemia was defined as serum low-density lipoprotein cholesterol levels $\ge$ 120 mg/dL, triglyceride levels $\ge$ 150 mg/dL, or high-density lipoprotein cholesterol levels < 40 mg/dL, in accordance with the Japan Atherosclerosis Society criteria, or the current use of lipid-lowering agents. Proteinuria was defined as a positive result using US-3500 or US-1200 analyzer and Uropaper III Eiken (Eiken Chemical, Co., Ltd., Tokyo, Japan). A positive result was urinary protein concentrations of $\ge$ 30 mg/dL, which are the same as those using the Albustix dipstick method. In the enrolled patients, there were missing data for the smoking status (20.7%), dyslipidemia (0.6%), and proteinuria (37.2%). There were no missing data for the other variables.

### Clinical outcomes

The outcome of this study was a composite of ESKD events, which was defined as an eGFR < 15 mL min^−1^ [1.73 m]^−2^, chronic dialysis, or renal transplantation.

### Statistical analysis

To select important variables for predicting ESKD, we evaluated the relative importance of 20 variables using a random forest model (randomForestSRC package, http://cran.r-project.org/web/packages/randomForestSRC). In this study, we also evaluated the predictive value of these variables for ESRD using the Cox proportional hazard model to confirm the results of the random forest model. In the Cox proportional hazard model, variables were standardized by subtracting the mean and dividing by the standard deviation, and they were then applied to the Cox proportional hazard model to compare the relative importance. Thus, we selected the important variables from both models, and then developed a sequential series of models using Cox proportional hazard analysis. We used Harrell’s concordance statistic (C-statistic) as a measure of discrimination for ESKD to evaluate the performance of the model^42^. Calibration was assessed using the Nam and D’Agostino $\chi$^2^ statistic to examine how closely each model’s predicted probabilities agreed with the observed ESKD outcomes^43^. The prediction risk calculated from a Cox proportional hazard model at time $t$ can be written as follows:

$${p}_{i}\left(t\right)=1-{S}_{i}\left(t\right)=1-{\mathrm{exp}(-{H}_{0}\left(t\right))}^{\mathrm{exp}(\sum_{p}{\beta }_{p}{x}_{ip})}$$

where ${S}_{i}\left(t\right)$ is a survivor function and ${H}_{0}\left(t\right)$ is a cumulative baseline hazard function^44^. The value of $(\sum_{p}{\beta }_{p}{x}_{ip})$ (${\beta }_{p}$, coefficient for variable $p$; ${x}_{ip},$variable $p$ for patient $i$) was defined as a prediction risk score, which was developed from the linear progression equation from the Cox hazard regression model. Given that the predicted absolute ESKD probability is clinically important, a nomogram was also built using the coefficients of the prediction model.

There were missing data for the smoking status, dyslipidemia, and proteinuria in this study. For our data imputation approach, we used the random forest imputation algorithm, called missForest (missForest package version 1.2, http://cran.r-project.org/web/packages/missForest)^45^. To confirm that our missForest prediction models were robust and not sensitive to missing data, we also used another imputation method, where we imputed the missing data by multiple imputation method (mice package version 3.14.0, https://cran.r-project.org/web/packages/mice/mice.pdf) in this analysis. The results in this method were similar to those in the missForest models (supplementary Table S1). Two-tailed *P* < 0.05 was defined as statistical significance. Data are presented as the mean ± standard deviation or the median (IQR). The significance of differences was determined by the chi-square test for categorical variables and the unpaired t test or the Mann–Whitney U test for continuous variables. We performed statistical analyses using R (version 3.6.3, https://www.r-project.org).

## Supplementary Information

Supplementary Information.

## Data Availability

The datasets generated and/or analyzed during the current study are available from the corresponding author on reasonable request.
