# Supplementary material for: A simplified prediction model for end-stage kidney disease in patients with diabetes
Source: Sci Rep. 2022 Jul 21;12:12482. doi: 10.1038/s41598-022-16451-5 (PMC9304378; doi:10.1038/s41598-022-16451-5)
Supplement: Supplementary file 1 — Supplementary Information. [file 41598_2022_16451_MOESM1_ESM.pdf]

Online-Only Supplementary Material

Table of Contents

Table S1 ………………………………………………………………. p. 2

Table S2 ………………………………………………………………. p. 3

Figure S1 ……………………………………………………………… p. 4

Table S1. Relative importance of variables for predicting end-stage kidney disease (ESKD) using the random forest model and the Cox proportional hazard model (multiple imputation method)

**Random forest model**<br>**C-statistic 0.905**	
**Upper 12 variables**	**Relative importance**
eGFR	0.098
Serum albumin	0.024
HbA1c	0.019
Proteinuria, positive	0.017
Serum uric acid	0.008
Serum bilirubin	0.007
Red blood cell count	0.005
ARB use	0.004
Age	0.002
Body mass index	0.001
Hypertension, positive	0.001
Thrombocyte count	0.000

**Cox proportional hazard model**<br>**C-statistic 0.905**			
**Upper 12 variables**	**HR**	**95% CI**	***P* value**
eGFR	0.245	0.195-0.308	<0.001
Serum bilirubin	0.557	0.417-0.744	<0.001
HbA1c	1.736	1.513-1.993	<0.001
Serum albumin	0.609	0.514-0.720	<0.001
Proteinuria, positive	1.624	1.291-2.043	<0.001
Age	0.707	0.591-0.846	<0.001
Serum uric acid	1.258	1.051-1.506	0.012
ARB use	1.228	1.013-1.490	0.037
GLP-1R agonist use	0.816	0.682-0.977	0.027
Gender, men	1.156	0.967-1.382	0.113
Hypertension, positive	0.883	0.672-1.161	0.374
Body mass index	0.887	0.735-1.071	0.213

* P<0.05. ARB, angiotensin II receptor blocker; GLP-1R, glucagon-like peptide 1 receptor.

Table S2. Characteristics of the subjects in the external validation cohort compared with those in the development cohort

**Variables**	**Validation cohort**	**Development cohort**	***P* value**
n	5153	2549	
Age, years, median (IQR)	48.0(42.0-54.0)	57.0 (47.0-63.0)	<0.001[a]
Gender, male, n (%)	4622 (89.7)	1432 (56.2)	<0.001[b]
HbA1c, %, median (IQR)	6.9 (6.4-7.9)	7.0 (6.5-8.1)	0.0831[a]
mmol/mol, median (IQR)	50.4 (45.3-60.6)	51.4 (46.3-62.6)	0.0831[a]
Serum albumin, mg/dL, mean (SD)	4.5 (0.4)	4.0 (0.5)	<0.001[c]
Serum bilirubin, mg/dL, median (IQR)	0.7 (0.6-1.0)	0.7 (0.5-0.9)	<0.001[a]
eGFR, mL min^{-1} [1.73 m]$^{-2}$, mean (SD)	80.6 (20.5)	81.8 (26.6)	0.0345[c]
Proteinuria, n (%)			
Positive,	984 (19.1)	475 (29.7)	0.6492[b]
Missing, n (%)	0	948 (37.2)	
Follow-up time, years,			
Median (IQR)	4.0 (3.9-4.6)	4.7 (2.3-8.8)	<0.001[a]
Mean (SD)	4.3 (1.8)	5.6 (3.7)	<0.001[c]
ESKD, n (%)	157 (3.1)	176 (6.2)	<0.001[c]
Time to ESKD, years,			
Median (IQR)	3.1 (1.6-4.5)	2.5 (0.9-4.8)	<0.001[a]
Mean (SD)	3.4 (2.2)	3.2 (2.9)	<0.001[c]

a Calculated by Mann Whitney test, b Calculated by c2 test, c Calculated by t test. ESRD, end-stage kidney disease. Abbreviation: eGFR, estimated glomerular filtration rate; HbA1c, hemoglobin A1c.

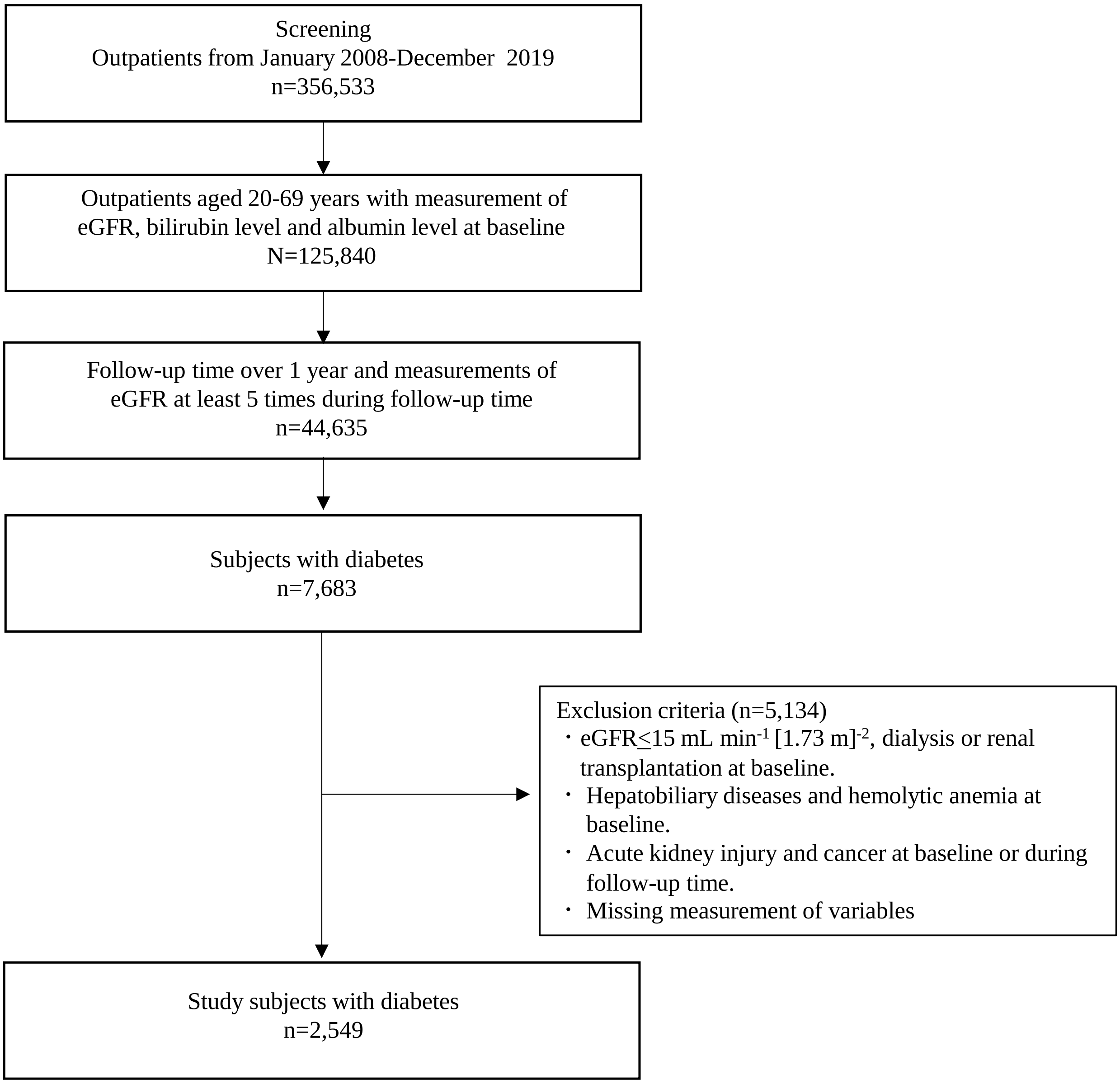

Fig. S1. Selection flow of study subjects
